# Supplementary material for: Energy efficiency to reduce residential electricity and natural gas use under climate change
Source: Nat Commun. 2017 May 15;8:14916. doi: 10.1038/ncomms14916 (PMC5440627; doi:10.1038/ncomms14916)
Supplement: Supplementary Information — Supplementary Figures, Supplementary Tables, Supplementary Notes, Supplementary Discussion, and Supplementary References [file ncomms14916-s1.pdf]

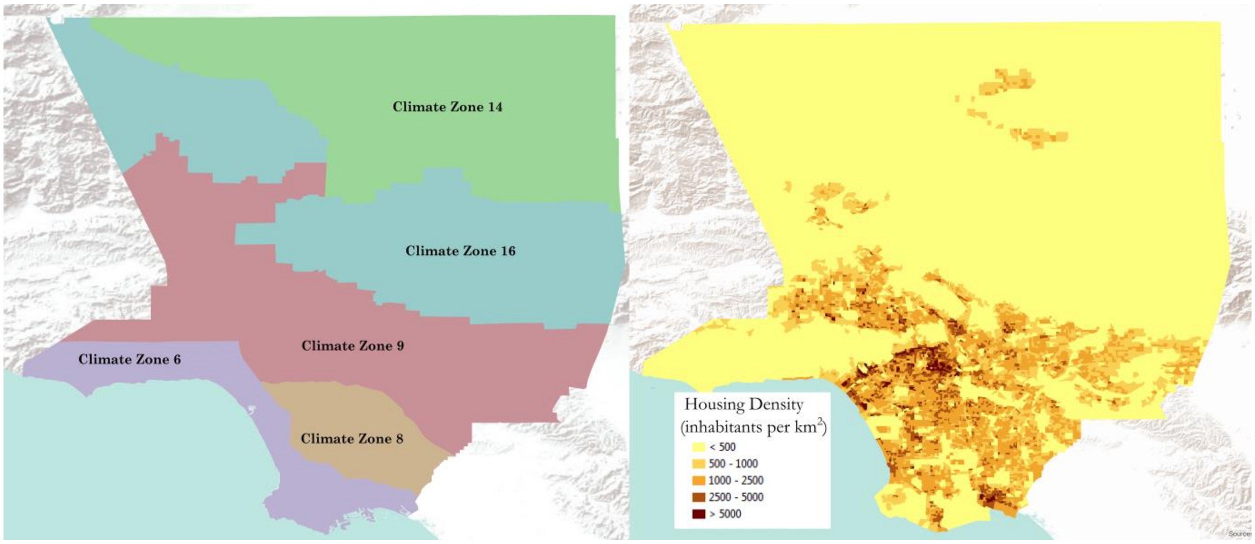

Supplementary Figure 1: Climate Zones and Population Density of Los Angeles County. LAC has five climate zones as designated by the California Energy Commission (left). Climate Zone 9 contains the City of Los Angeles and is the most densely population area of LAC (right).

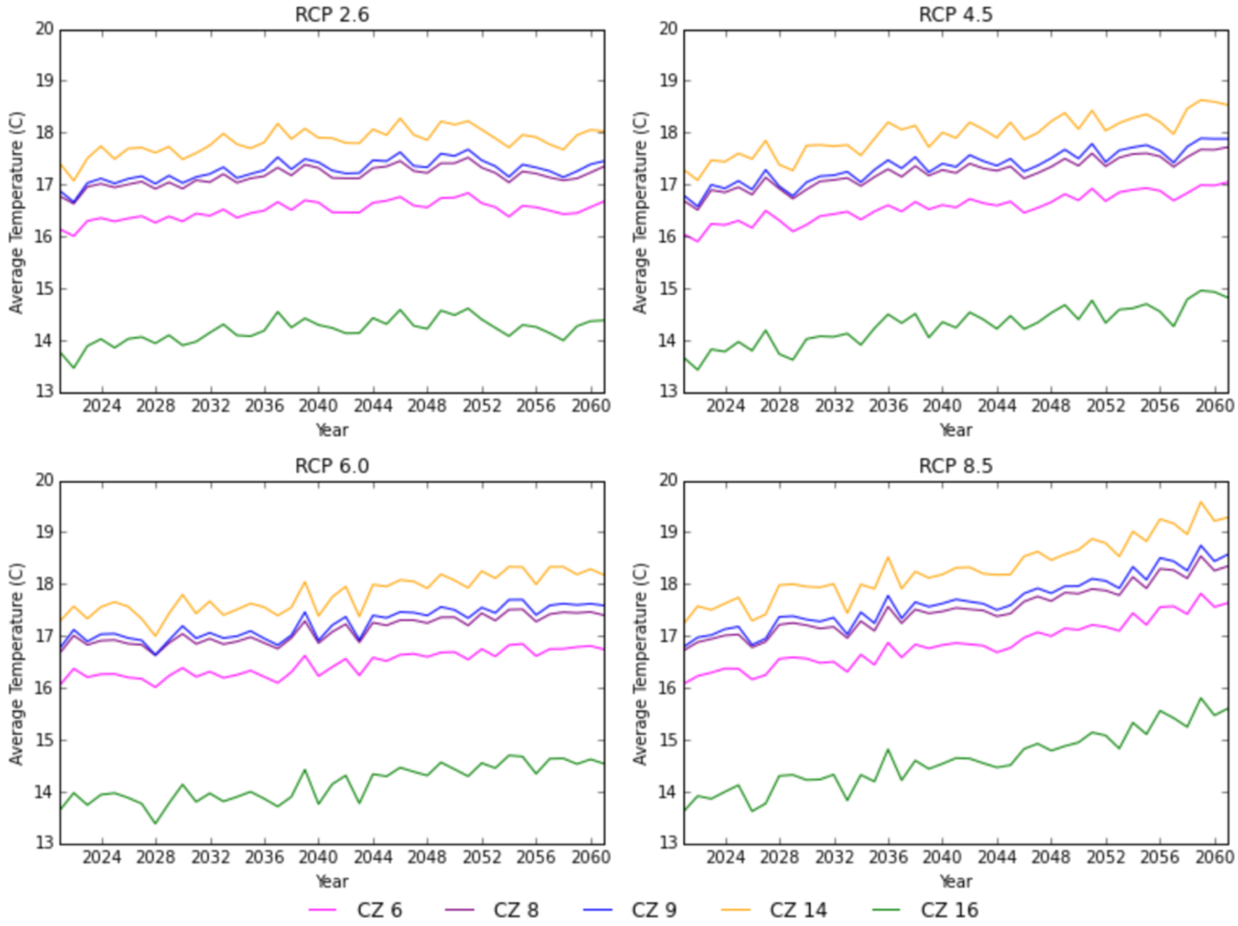

Supplementary Figure 2: Projected Temperature Increases. For each RCP we graph the average annual temperature from the GCMs by climate zone.

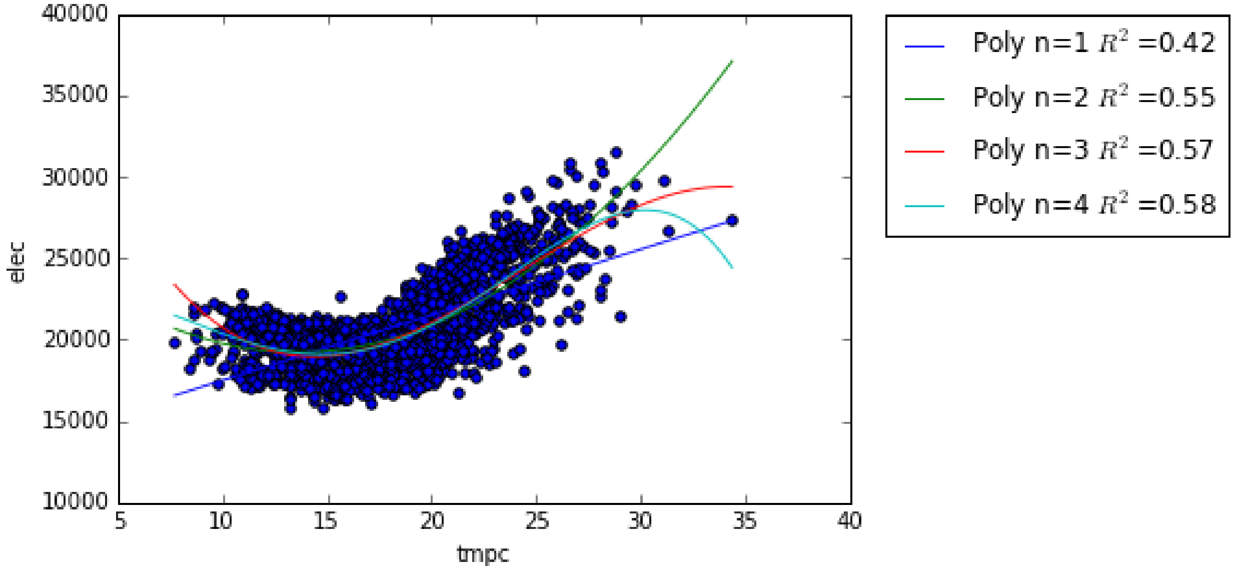

Supplementary Figure 3: Scatterplot and Regression Models between Temperature (tmpc – daily average temperature in degrees Celsius) and Residential Electricity Consumption (elec – LADWP daily residential electricity use in kWh).

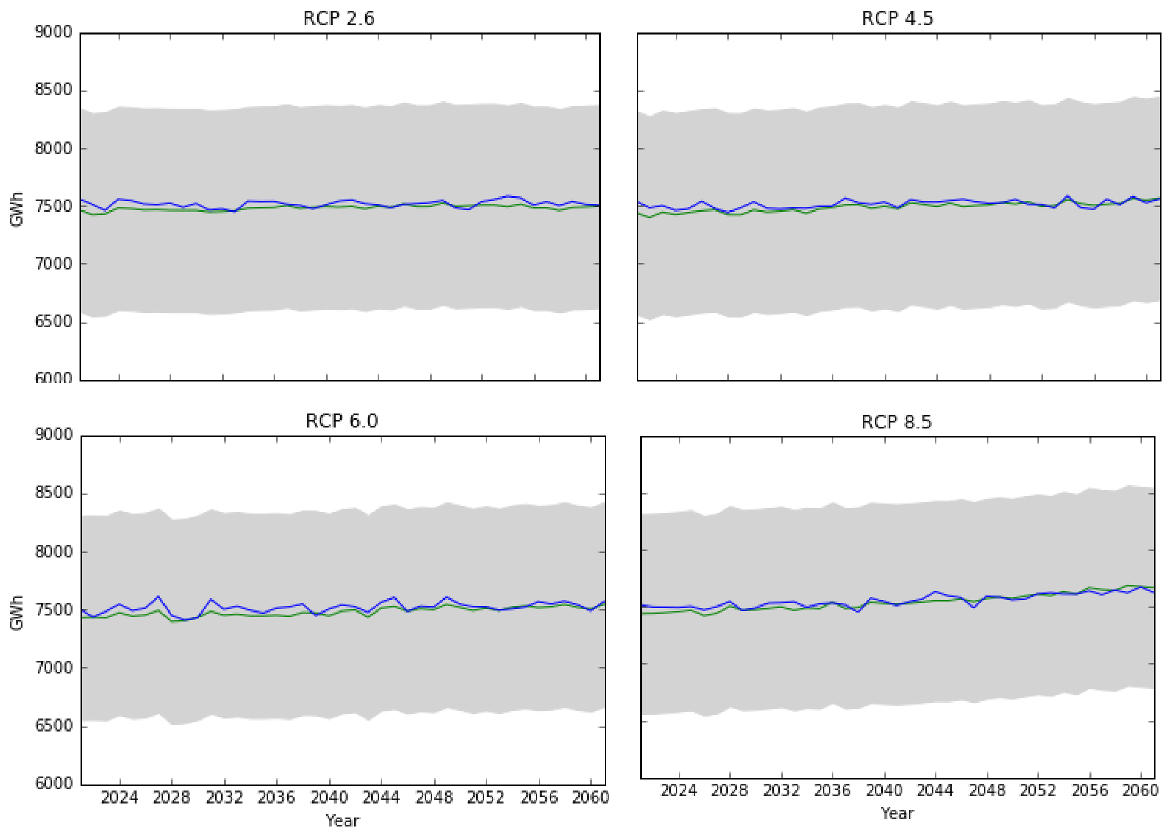

Supplementary Figure 4: Regression vs Archetype Model. The regression model forecasts are in green with a grey 95% confidence interval, and the archetype forecasts are blue.

Supplementary Table 1: RCP 8.5 - Electricity End Use Results Summary by Scenario, 2020 and 2060 (GWh)

	Base - 2020	Scenario 1 - 2060	Scenario 2 - 2060	Scenario 3 - 2060	Scenario 4 - 2060
Heating/Cooling	*4,458*	*10,723*	*17,640*	*14,965*	*11,923*
Water Heating	*272*	*206*	*2,086*	*1,285*	*316*
Main Fridge	*2,702*	*2,208*	*2,208*	*1,661*	*1,141*
TV	*2,735*	*2,211*	*2,211*	*1,649*	*1,133*
Computer	*1,901*	*1,593*	*1,593*	*1,228*	*844*
Pool	*1,465*	*1,562*	*1,562*	*1,447*	*1,447*
2nd Fridge	*1,076*	*918*	*918*	*721*	*495*
3rd Fridge	*47*	*37*	*37*	*27*	*18*
Freezer	*533*	*440*	*440*	*341*	*234*
Microwave	*383*	*236*	*236*	*147*	*128*
Oven/Range	*266*	*354*	*354*	*306*	*306*
Lights	*3,193*	*2,129*	*2,129*	*2,027*	*1,854*
Exterior Lights	*1,664*	*1,247*	*1,331*	*1,164*	*1,049*
Miscellaneous	*1,915*	*9,104*	*9,104*	*8,697*	*9,022*
**Total**	***22,610***	***32,969***	***41,848***	***35,665***	***29,910***

Supplementary Table 2: RCP 8.5 – Natural Gas End Use Results Summary by Scenario (Joules)

	Base - 2020	Scenario 1 - 2060	Scenario 2 - 2060	Scenario 3 - 2060	Scenario 4 - 2060
Heating/Cooling	*13.5*	*19.3*	*3.1*	*0.3*	*0.5*
Water Heating	*14.0*	*2.5*	*9.3*	*6.4*	*2.9*
Oven/Range	*8.0*	*2.2*	*2.2*	*2.0*	*2.0*
Pool	*2.7*	*5.2*	*5.3*	*4.9*	*4.9*
Miscellaneous	*1.7*	*1.7*	*1.7*	*1.7*	*1.7*
**Total**	***39.9***	***31.0***	***21.6***	***15.3***	***12.1***

Supplementary Table 3: RCP 6.0 - Electricity End Use Results Summary by Scenario (GWh)

	Base - 2020	Scenario 1 - 2060	Scenario 2 - 2060	Scenario 3 - 2060	Scenario 4 - 2060
Heating/Cooling	*4,458*	*10,169*	*16,815*	*14,221*	*11,308*
Water Heating	*272*	*206*	*2,086*	*1,285*	*316*
Main Fridge	*2,702*	*2,208*	*2,208*	*1,661*	*1,141*
TV	*2,735*	*2,211*	*2,211*	*1,649*	*1,133*
Computer	*1,901*	*1,593*	*1,593*	*1,228*	*844*
Pool	*1,465*	*1,562*	*1,562*	*1,447*	*1,447*
2nd Fridge	*1,076*	*918*	*918*	*721*	*495*
3rd Fridge	*47*	*37*	*37*	*27*	*18*
Freezer	*533*	*440*	*440*	*341*	*234*
Microwave	*383*	*236*	*236*	*147*	*128*
Oven/Range	*266*	*354*	*354*	*306*	*306*
Lights	*3,193*	*2,129*	*2,129*	*2,027*	*1,854*
Exterior Lights	*1,664*	*1,247*	*1,331*	*1,164*	*1,049*
Miscellaneous	*1,915*	*9,104*	*9,104*	*8,697*	*9,022*
**Total**	***22,610***	***32,414***	***41,023***	***34,922***	***29,295***

Supplementary Table 4: RCP 6.0 – Natural Gas End Use Results Summary by Scenario (Joules)

	Base - 2020	Scenario 1 - 2060	Scenario 2 - 2060	Scenario 3 - 2060	Scenario 4 - 2060
Heating/Cooling	*14.1*	*21.1*	*3.1*	*0.3*	*0.5*
Water Heating	*14.0*	*2.5*	*9.3*	*6.4*	*2.9*
Oven/Range	*8.0*	*2.2*	*2.2*	*2.0*	*2.0*
Pool	*2.7*	*5.2*	*5.3*	*4.9*	*4.9*
Miscellaneous	*1.7*	*1.7*	*1.7*	*1.7*	*1.7*
**Total**	***40.5***	***32.8***	***21.6***	***15.3***	***12.1***

Supplementary Table 5: RCP 4.5 - Electricity End Use Results Summary by Scenario (GWh)

	Base - 2020	Scenario 1 - 2060	Scenario 2 - 2060	Scenario 3 - 2060	Scenario 4 - 2060
Heating/Cooling	*4,458*	*10,133*	*16,740*	*14,161*	*11,265*
Water Heating	*272*	*206*	*2,086*	*1,285*	*316*
Main Fridge	*2,702*	*2,208*	*2,208*	*1,661*	*1,141*
TV	*2,735*	*2,211*	*2,211*	*1,649*	*1,133*
Computer	*1,901*	*1,593*	*1,593*	*1,228*	*844*
Pool	*1,465*	*1,562*	*1,562*	*1,447*	*1,447*
2nd Fridge	*1,076*	*918*	*918*	*721*	*495*
3rd Fridge	*47*	*37*	*37*	*27*	*18*
Freezer	*533*	*440*	*440*	*341*	*234*
Microwave	*383*	*236*	*236*	*147*	*128*
Oven/Range	*266*	*354*	*354*	*306*	*306*
Lights	*3,193*	*2,129*	*2,129*	*2,027*	*1,854*
Exterior Lights	*1,664*	*1,247*	*1,331*	*1,164*	*1,049*
Miscellaneous	*1,915*	*9,104*	*9,104*	*8,697*	*9,022*
**Total**	***22,610***	***32,378***	***40,949***	***34,862***	***29,251***

Supplementary Table 6: RCP 4.5 – Natural Gas End Use Results Summary by Scenario (Joules)

	Base - 2020	Scenario 1 - 2060	Scenario 2 - 2060	Scenario 3 - 2060	Scenario 4 - 2060
Heating/Cooling	*14.5*	*20.0*	*3.1*	*0.3*	*0.5*
Water Heating	*14.0*	*2.5*	*9.3*	*6.4*	*2.9*
Oven/Range	*8.0*	*2.2*	*2.2*	*2.0*	*2.0*
Pool	*2.7*	*5.2*	*5.3*	*4.9*	*4.9*
Miscellaneous	*1.7*	*1.7*	*1.7*	*1.7*	*1.7*
**Total**	***40.9***	***31.7***	***21.6***	***15.3***	***12.1***

Supplementary Table 7: RCP 2.6 - Electricity End Use Results Summary by Scenario, 2020 and 2060 (GWh)

	Base - 2020	Scenario 1 - 2060	Scenario 2 - 2060	Scenario 3 - 2060	Scenario 4 - 2060
Heating/Cooling	*4,458*	*9,675*	*16,044*	*13,550*	*10,763*
Water Heating	*272*	*206*	*2,086*	*1,285*	*316*
Main Fridge	*2,702*	*2,208*	*2,208*	*1,661*	*1,141*
TV	*2,735*	*2,211*	*2,211*	*1,649*	*1,133*
Computer	*1,901*	*1,593*	*1,593*	*1,228*	*844*
Pool	*1,465*	*1,562*	*1,562*	*1,447*	*1,447*
2nd Fridge	*1,076*	*918*	*918*	*721*	*495*
3rd Fridge	*47*	*37*	*37*	*27*	*18*
Freezer	*533*	*440*	*440*	*341*	*234*
Microwave	*383*	*236*	*236*	*147*	*128*
Oven/Range	*266*	*354*	*354*	*306*	*306*
Lights	*3,193*	*2,129*	*2,129*	*2,027*	*1,854*
Exterior Lights	*1,664*	*1,247*	*1,331*	*1,164*	*1,049*
Miscellaneous	*1,915*	*9,104*	*9,104*	*8,697*	*9,022*
**Total**	***22,610***	***31,920***	***40,253***	***34,251***	***28,750***

Supplementary Table 8: RCP 2.6 – Natural Gas End Use Results Summary by Scenario (Joules)

	Base - 2020	Scenario 1 - 2060	Scenario 2 - 2060	Scenario 3 - 2060	Scenario 4 - 2060
Heating/Cooling	*14.3*	*20.6*	*3.1*	*0.3*	*0.5*
Water Heating	*14.0*	*2.5*	*9.3*	*6.4*	*2.9*
Oven/Range	*8.0*	*2.2*	*2.2*	*2.0*	*2.0*
Pool	*2.7*	*5.2*	*5.3*	*4.9*	*4.9*
Miscellaneous	*1.7*	*1.7*	*1.7*	*1.7*	*1.7*
**Total**	***40.7***	***32.3***	***21.6***	***15.3***	***12.1***

Supplementary Table 9: Climate Zone 6 – HVAC percent of end use in 2020 and 2060 by scenario and RCP

	2020 - S1	*2060 - S1*	*2060 - S2*	*2060 - S3*	*2060 - S4*
8.5	13%	12%	58%	53%	48%
6.0	13%	12%	57%	51%	46%
4.5	13%	12%	56%	51%	46%
2.6	13%	11%	54%	49%	44%

Supplementary Table 10: Climate Zone 8 – HVAC percent of end use in 2020 and 2060 by scenario and RCP

	2020 - S1	*2060 - S1*	*2060 - S2*	*2060 - S3*	*2060 - S4*
8.5	13%	29%	39%	37%	35%
6.0	13%	26%	36%	34%	32%
4.5	13%	27%	36%	35%	33%
2.6	13%	25%	35%	33%	31%

Supplementary Table 11: Climate Zone 9 – HVAC percent of end use in 2020 and 2060 by scenario and RCP

	2020 - S1	*2060 - S1*	*2060 - S2*	*2060 - S3*	*2060 - S4*
8.5	21%	34%	43%	42%	40%
6.0	21%	33%	42%	41%	39%
4.5	21%	33%	42%	41%	39%
2.6	21%	32%	41%	40%	38%

Supplementary Table 12: Climate Zone 14 – HVAC percent of end use in 2020 and 2060 by scenario and RCP

	2020 - S1	*2060 - S1*	*2060 - S2*	*2060 - S3*	*2060 - S4*
8.5	30%	42%	51%	49%	47%
6.0	30%	40%	50%	47%	46%
4.5	30%	40%	49%	47%	45%
2.6	30%	39%	48%	46%	44%

Supplementary Table 13: Climate Zone 16 – HVAC percent of end use in 2020 and 2060 by scenario and RCP

	2020 - S1	*2060 - S1*	*2060 - S2*	*2060 - S3*	*2060 - S4*
8.5	28%	40%	50%	50%	47%
6.0	28%	40%	50%	50%	47%
4.5	28%	39%	49%	49%	46%
2.6	28%	39%	49%	48%	46%

Supplementary Table 14: Base Model HVAC Technologies. HVAC Technologies are weighted based on their prevalence within each prototype category to capture the variability in the stock. Since the presence of appliances in buildings impacts thermal loading, we include a typical set of appliances which remained constant across all the HVAC simulations, but utilized only the heating and cooling end use consumption from the HVAC simulations.

Category	Efficiency
Furnace	*Electric 100% AFUE*
Furnace	*Gas 64% AFUE*
Furnace	*Gas 68% AFUE*
Furnace	*Gas 80% AFUE*
Furnace	*Propane 64% AFUE*
Furnace	*Propane 68% AFUE*
Furnace	*Propane 80% AFUE*
Boiler	*Electric*
Boiler	*NG, Forced Draft, 72% AFUE*
Boiler	*NG, Forced Draft, 76% AFUE*
Boiler	*NG, Forced Draft, 80% AFUE*
Boiler	*Propane, Forced Draft, 72% AFUE*
Boiler	*Propane, Forced Draft, 76% AFUE*
Boiler	*Propane, Forced Draft, 80% AFUE*
Electric Baseboard	*100% Efficiency*
Air-Source Heat Pump	*SEER 8, 6 HSPF*
Air-Source Heat Pump	*SEER 10, 6.2 HSPF*
Air-Source Heat Pump	*SEER 13, 7.7 HSPF*
Air-Source Heat Pump	*SEER 14, 8.2 HSPF*
Air-Source Heat Pump	*SEER 15, 8.5 HSPF*
Mini-Split Heat Pump	*SEER 14*
Central A/C	*SEER 8*
Central A/C	*SEER 10*
Central A/C	*SEER 13*
Central A/C	*SEER 14*
Central A/C	*SEER 15*
Room A/C	*EER 8.5*
Room A/C	*EER 9.8*

Supplementary Table 15: LAC Climate Zones – Heating Degree Days and Cooling Degree Days

Climate Zone	*HDD*	*CDD*
6	1460	730
8	1290	1300
9	1150	1540
14	2704	1998
16	4300	1040

Supplementary Table 16: EPW Parameters and Data Sources (Note: Fields marked "N/A" are currently not used in EnergyPlus calculations)

EPW Name	EPW Parameter	Data Sources
N1-N5	*Year, month, day, hour minute*	*Self-Produced*
A1	*Uncertainty Flags*	*N/A*
N6	*Dry bulb temperature (℃)*	*IEM ASOS*
N7	*Dew point temperature (℃)*	*IEM ASOS*
N8	*Relative humidity (%)*	*IEM ASOS*
N9	*Atmospheric pressure (Pa)*	*IEM ASOS*
N10	*Extraterrestrial horizontal radiation (Wh/m^2)*	*N/A*
N11	*Extraterrestrial direct normal radiation (Wh/m^2)*	*N/A*
N12	*Horizontal infrared radiation from sky (Wh/m^2)*	*SolarAnywhere*
N13	*Global horizontal radiation (Wh/m^2)*	*N/A*
N14	*Direct normal radiation (Wh/m^2)*	*SolarAnywhere*
N15	*Diffuse horizontal radiation (Wh/m^2)*	*SolarAnywhere*
N16	*Global horizontal illuminance (lux)*	*N/A*
N17	*Direct normal illuminance (lux)*	*N/A*
N18	*Diffuse horizontal illuminance (lux)*	*N/A*
N19	*Zenith luminance (Cd/m^2)*	*N/A*
N20	*Wind direction (degrees)*	*IEM ASOS*
N21	*Wind speed (m/s)*	*IEM ASOS*
N22	*Total sky cover (tenths of sky)*	*N/A*
N23	*Opaque sky cover (tenths of sky)*	*N/A*
N24	*Visibility (km)*	*IEM ASOS*
N25	*Ceiling height*	*N/A*
N26	*Present weather observation*	*Self-Produced*
N27	*Present weather codes*	*Self-Produced*
N28	*Precipitable water (mm)*	*N/A*
N29	*Aerosol optical depth*	*N/A*
N30	*Snow depth*	*Not Used*
N31	*Days since last snowfall*	*N/A*
N32	*Albedo*	*N/A*
N33	*Liquid Precipitation Depth*	*IEM ASOS*
N34	*Liquid Precipitation Quantity*	*N/A*

Supplementary Table 17: Model End use and Total Compared to LADWP Data. The final calibrated model predicts total annual electricity use for LADWP within 5% of the expected value

End use	Model	LADWP	Variation
Other	*23.7%*	*24.7%*	*-1.0%*
Lighting	*14.8%*	*15.0%*	*-0.2%*
Fridge	*17.8%*	*13.9%*	*-0.5%*
TV	*13.1%*	*12.6%*	*0.5%*
PC	*8.7%*	*9.1%*	*-0.4%*
Pool	*6.1%*	*6.3%*	*-0.2%*
Cooling	*6.4%*	*6.0%*	*0.4%*
Heating	*0.1%*	*0.1%*	*0.0%*
Freezer	*2.0%*	*2.1%*	*-0.1%*
Microwave	*1.8%*	*2.0%*	*-0.2%*
Water Heating	*1.5%*	*1.6%*	*-0.1%*
Range	*1.2%*	*1.2%*	*0.0%*
Fans	*2.6%*	*1.1%*	*1.5%*
Total (GWh)	*7,488*	*7,190*	*298*
Percent in model	*104.1%*	*100.0%*	*4.1%*

Supplementary Table 18: Number of Runs Utilized from Each Climate Model

Model ID	Project Code	RCP2.6	RCP 4.5	RCP 6.0	RCP8.5
Access1-0	*ACC*	-	*1*	-	*1*
BCC-CSM1-1	*BCC*	*1*	-	*1*	-
CCSM4	*CCS*	*2*	*2*	*2*	*2*
CESM1-BGC	*CES*	-	-	-	-
CNRM-CM5	*CNR*	-	*1*	-	*1*
GFDL-CM3	*GFC*	*1*	-	*1*	-
GFDL-ESM2M	*GFG*	*1*	*1*	*1*	*1*
GFDL-ESM2M	*GFM*	-	-	*1*	-
INMCM4	*INM*	-	*1*	-	*1*
IPSL-CM5A-LR	*IPL*	-	-	*1*	-
IPSL-CM5A-MR	*IPM*	*1*	-	*1*	-
MIROC5	*MIR*	*1*	*1*	-	*1*
MPI-ESM-LR	*MPL*	*3*	*3*	-	*3*
MRI-CGCM3	*MRI*	-	-	*1*	-
NORESM1-M	*NOR*	-	-	*1*	-

Supplementary Table 19: Cumulative Housing Growth in LAC

Year	Growth
2010	*0.0%*
2015	*3.3%*
2020	*9.5%*
2025	*12.3%*
2030	*15.9%*
2035	*17.9%*
2040	*20.0%*
2045	*21.4%*
2050	*22.2%*
2055	*22.5%*
2060	*22.5%*

Supplementary Table 20: Additional HVAC Technologies for Forecasting

Category	Efficiency
Furnace	*Gas 90% AFUE*
Furnace	*Gas 98% AFUE*
Boiler	*NG, Forced Draft, 85% AFUE*
Boiler	*NG, Forced Draft, 98% AFUE*
Air-Source Heat Pump	*SEER 19*
Air-Source Heat Pump	*SEER 22*
Mini-Split Heat Pump	*SEER 26*
Central A/C	*SEER 17*
Central A/C	*SEER 18*
Central A/C	*SEER 21*
Room A/C	*EER 10.7*

Supplementary Table 21: Appliance Categories. To save on computation time, we model appliances independently of HVAC and building shell, but with a weighted approach for each to capture the range of technologies

Category	Efficiency
Water Heater	*Electric Standard*
Water Heater	*Electric Tankless*
Water Heater	*Natural Gas Standard*
Water Heater	*Natural Gas Tankless*
Water Heater	*Natural Gas Premium*
Water Heater	*Propane Standard*
Water Heater	*Propane Tankless*
Water Heater	*Heat Pump*
Water Heater	*Solar Water Heating*
Refrigerator	*25 cu ft, EF=6.5, side freezer*
Refrigerator	*18 cu ft, EF=6.9, top freezer*
Refrigerator	*21 cu ft, EF=6.7, bottom freezer*
Refrigerator	*25 cu ft, EF=13.8, side freezer*
Refrigerator	*18 cu ft, EF=14.1, top freezer*
Refrigerator	*21 cu ft, EF=13.6, bottom freezer*
Refrigerator	*25 cu ft, EF=4.4, side freezer*
Refrigerator	*18 cu ft, EF=4.4, top freezer*
Refrigerator	*21 cu ft, EF=4.5, bottom freezer*
Refrigerator	*25 cu ft, EF=10.8, side freezer*
Refrigerator	*18cu ft, EF=10.5, top freezer*
Refrigerator	*21 cu ft, EF=10.2, bottom freezer*
Refrigerator	*25 cu ft, EF=15.7, side freezer*
Refrigerator	*18 cu ft, EF=15.9, top freezer*
Refrigerator	*21 cu ft, EF=15.9, bottom freezer*
TVs	*Standard TVs*
TVs	*Plasma TVs*
TVs	*Large LCD TVs*
TVs	*Small LCD TVs*
Computers	*Desktop*
Computers	*Laptop*
Pool	*No Heat Pool*
Pool	*Electric Pool*
Pool	*Natural Gas Pool*
Freezer	*16 cu ft. EF=13, chest, 11-20 years*
Freezer	*18 cu ft. EF=9, upright, 11-20 years*
Freezer	*16 cu ft. EF=24, chest, 2-7 years*
Freezer	*18 cu ft. EF=16, upright, 2-7 years*
Freezer	*16 cu ft. EF=10, chest, 21+ years*
Freezer	*18 cu ft. EF=6, upright, 21+ years*
Freezer	*18 cu ft. EF=18, chest, 8-10 years*
Freezer	*16 cu ft. EF=12, upright, 8-10 years*
Freezer	*16 cu ft. EF=27, chest, <2 years*
Freezer	*18 cu ft. EF=18, upright, <2 years*
Microwave	*0-5 years*
Microwave	*6-10 years*
Microwave	*11-15 years*
Microwave	*15+ years*
Cooking Range / Oven	*Electric*
Cooking Range / Oven	*Conventional Gas*
Cooking Range / Oven	*Conventional Propane*

Supplementary Table 22: Incremental Cooling Cost. For heating and cooling we develop a weighted average cost of a heating or cooling unit for Scenarios 2 and 4. The difference in the scenarios within the RCP is the incremental cost needed for the CCE calculation. We pull the cost of purchasing and installing each type of unit from www.homeadvisor.com which contains user-reported data on home repairs. We weight the cost based upon the count of each type of HVAC system in 2060.

Type	Cost (2015$)	S4_8.5	S2_8.5	S4_6.0	S2_6.0	S4_4.5	S2_4.5	S4_2.6	S2_2.6
ASHP - SEER8	*$5,266*	*0*	*0*	*0*	*0*	*0*	*0*	*0*	*0*
ASHP - SEER10	*$5,266*	*0*	*0*	*0*	*0*	*0*	*0*	*0*	*0*
ASHP - SEER13	*$5,266*	*0*	*0*	*0*	*0*	*0*	*0*	*0*	*0*
ASHP - SEER14	*$5,266*	*0*	*369,191*	*0*	*369,191*	*0*	*369191*	*0*	*369191*
ASHP - SEER15	*$5,266*	*1*	*277,593*	*1*	*277,593*	*1*	*277593*	*1*	*277593*
ASHP - SEER19	*$5,266*	*0*	*185,014*	*0*	*185,014*	*0*	*185,014*	*0*	*185,014*
ASHP - SEER22	*$5,266*	*3,547,753*	*371,380*	*3,490,265*	*311,541*	*3,501,361*	*323,101*	*3,463,156*	*283,110*
MSHP - SEER14	*$5,266*	*0*	*36,315*	*0*	*36,315*	*0*	*36,315*	*0*	*36,315*
MSHP - SEER26	*$5,266*	*0*	*36,315*	*0*	*36,315*	*0*	*36,315*	*0*	*36,315*
CAC - SEER8	*$5,240*	*0*	*0*	*0*	*0*	*0*	*0*	*0*	*0*
CAC - SEER10	*$5,240*	*0*	*0*	*0*	*0*	*0*	*0*	*0*	*0*
CAC - SEER13	*$5,240*	*0*	*0*	*0*	*0*	*0*	*0*	*0*	*0*
CAC - SEER14	*$5,240*	*0*	*513,204*	*0*	*513,204*	*0*	*513,204*	*0*	*513,204*
CAC - SEER15	*$5,240*	*146*	*550,543*	*146*	*550,543*	*146*	*550,543*	*146*	*550,543*
CAC - SEER17	*$5,240*	*0*	*370,036*	*0*	*370,036*	*0*	*370,036*	*0*	*370,036*
CAC - SEER18	*$5,240*	*0*	*189,668*	*0*	*189,668*	*0*	*189,668*	*0*	*189,668*
CAC - SEER21	*$5,240*	*0*	*180,368*	*0*	*180,368*	*0*	*180,368*	*0*	*180,368*
RAC - EER8.5	*$2,400*	*0*	*0*	*0*	*0*	*0*	*0*	*0*	*0*
RAC - EER9.8	*$2,400*	*512*	*219,855*	*512*	*219,855*	*512*	*219,855*	*512*	*219,855*
RAC - EER10.7	*$2,400*	*0*	*219,287*	*0*	*219,287*	*0*	*219,287*	*0*	*219,287*
**Average Cost**		***$5,265***	***$4,895***	***$5,266***	***$4,889***	***$5,266***	***$4,890***	***$5,266***	***$4,885***

Supplementary Table 23: Incremental Heating Cost

Type	Cost (2015$)	S4	S2
Furnace - Electric	*$2,600*	*11,646*	*476,215*
Boiler - Electric	*$5,104*	*3,257*	*70,859*
Electric Baseboard	*$637*	*73,723*	*274,436*
ASHP - SEER8	*$5,266*	*0*	*0*
ASHP - SEER10	*$5,266*	*0*	*0*
ASHP - SEER13	*$5,266*	*0*	*0*
ASHP - SEER14	*$5,266*	*0*	*977,730*
ASHP - SEER15	*$5,266*	*2*	*734,222*
ASHP - SEER19	*$5,266*	*0*	*489,173*
ASHP - SEER22	*$5,266*	*3,578,435*	*244,740*
MSHP - SEER14	*$5,266*	*0*	*183,981*
MSHP - SEER26	*$5,266*	*0*	*183,981*
**Average Cost**		***$5,164***	***$4,564***

Supplementary Table 24: Incremental Cost Water Heating. Similar to HVAC, for water heating, we develop a weighted cost of equipment for each scenario. The source of the cost data for this portion is a 2009 market analysis from the U.S. Department of Energy[1].

Type	Cost (2009$)	S4	S2
Electric Standard	*$650*	*0*	*3,089,743*
Electric Tankless	*$1,255*	*1,078,256*	*277,178*
50 gal HP @ 125F	*$1,500*	*1,437,674*	*154,649*
Solar Water Heating (2)	*$3,200*	*1,078,256*	*72,615*
**Average Cost**		***$1,937***	***$785***

Supplementary Table 25: Incremental Cost Refrigerators. In our model for refrigerators and freezers, we do not change the count of each type of appliance in the future forecast. Instead, we make the average electricity consumption of each appliance end use category systematically more efficient. As a proxy for the cost difference of this efficiency improvement, we use the difference in cost between standard refrigerators and freezers from 2007 with the Energy Star models of that same year, based upon a report from the U.S. Department of Energy[2].

	Standard (2007$)	Energy Star (2007$)	Count
Side Freezer	*$1,128*	*$1,336*	*2,001,626*
Top Freezer	*$660*	*$663*	*1,709,038*
Bottom Freezer	*$1,285*	*$1,254*	*391,541*
**Weighted Difference**			***$100***

Supplementary Table 26: Incremental Cost Freezers

	Standard (2007$)	Energy Star (2007$)	Count
Upright	*$495*	*$602*	*190,613*
Chest	*$352*	*$473*	*90,841*
**Weighted Difference**			***$112***

Supplementary Table 27: Efficiency Savings Between Scenarios 2 and 4 in 2060, RCP 8.5. The demand difference in the year 2060 subdivided by end use consumption to identify the sources of the efficiency savings.

	Change GWh	Savings Percentage	Cost to Conserve (2010 ¢/kWh)[3]	Estimated Savings (2010 $)
Heating/Cooling	*-3845.9*	*38.5%*	*6.6*	*-$499,813,541*
Water Heater	*-1770.1*	*17.7%*	*23.8*	*$74,937,468*
Refrigerators	*-1507.8*	*15.1%*	*2.7*	*-$254,275,236*
TV	*-1078.0*	*10.8%*	*0.9*	*-$201,435,311*
Computer	*-749.6*	*7.5%*	*4.5*	*-$113,313,268*
Lights	*-556.8*	*5.6%*	*1.4*	*-$101,359,332*
Freezer	*-206.3*	*2.1%*	*1.4*	*-$37,627,345*
Pool	*-114.9*	*1.2%*	*2.5*	*-$19,691,366*
Microwave	*-107.8*	*1.1%*	*1.8*	*-$19,161,662*
Oven/Range	*-48.1*	*0.5%*	*7.1*	*-$6,014,756*
**Total**	***-9985.3***	***100.0%***		***-$1,177,754,349***

SUPPLEMENTARY NOTE 1: ARCHETYPE DEVELOPMENT

In Los Angeles County, the climate varies greatly between coastal and inland regions, so we differentiate archetypes based upon five climate zones. We provide a brief description of each climate zone. Climate Zone 6 includes coastal beaches and low lying coastal land along the southern California coast. The ocean regulates temperature in the summer and winter, keeping the climate mild. Climate Zone 8 is further inland, but is regulated by marine air, keeping the high and low temperatures more moderate than further inland zones. Summers are generally warmer and winters cooler than on the coast, so more heating and cooling are necessary. Climate Zone 9 has influence of both coastal and interior weather patterns. Cool moist air arrives from the ocean and hot dry air from further inland. Climate Zone 14 is a high desert climate characterized by large temperature

swings without the mediating influence of the ocean. Climate Zone 16 is about 5,000 feet in elevation and semi-arid. This is a colder climate than the other four in LAC. A summary of the heating degree days (HDD) and cooling degree days (CDD) is given in Supplementary Table 15. These are the summation of degrees above or below the
reference temperature (80˚F or 65 ˚F, respectively) per day. Climate information is adapted from the Pacific Energy Center's Guide to California Climate Zones and Bioclimatic Design[4].

SUPPLEMENTARY NOTE 2: COST OF CONSERVED ENERGY

In RCP 8.5 – Scenario 4, each household would save approximately $300 (2010$) in 2060 compared to Scenario 2, including the added cost of appliance upgrades without subsidies or rebates. This means that including the cost of equipment upgrades, households will save money on average from installing more efficient appliances in the home. With current electricity prices, all efficiency upgrades are cost savings with the exception of water heaters.

Using our modeled results, we can estimate the average cost savings to consumers using a concept known as cost of conserved energy (CCE). CCE quantifies the amount of money that needs to be spent on a specific intervention to save a unit of electricity:

$$CCE = \frac{I*d}{(1-(1+d)^{-n})*S} \quad \text{(Supplementary Equation 1)}$$

CCE = Cost of conserved energy ($/kWh)

I = Incremental Cost ($)

d= Discount Rate

S = Annual energy savings (kWh/yr)

n=lifetime of mitigation option (yrs)

We develop CCE estimates for each of the 10 end-uses in Supplementary **Table 27**
based on the difference in energy consumption between Scenario 2 and Scenario
4 under RCP 8.5. These CCE values can then be used to calculate the cost to homeowners of the efficiency differences between the two scenarios. For the highest contributing end uses to the total savings (HVAC, water heating, and refrigerators/freezers), we develop our own CCE estimates. These end uses comprise ~70% of the energy savings. For the other end uses, we use CCEs developed for a National Academy of Sciences energy efficiency study[3]. In the following sections, we discuss the development of the incremental cost estimates for heating, cooling, water heating, and refrigerators/freezers.

We use the CCE for each of the end use types to estimate the average savings to each household in LAC in 2060, based upon a method in the National Academy of Sciences study[3]. We calculate the total cost to the consumer by multiplying the CCE for each end use by the electricity savings in 2060, and then compare this to the amount that that electricity would have cost had it been purchased from the utility company. All costs are adjusted with inflation to 2010$, as this is the base year of the model. We use a discount rate of 7% to remain consistent with the CCE values from the National Academies study. In February 2015, the average price of electricity in Los Angeles County was 21.6 ¢/kWh[5]. Converting this to 2010 dollars, the equivalent price would be 19.6 ¢/kWh. We assume that the cost of electricity remains constant through 2060. This is approximately $300 (2010$) per household savings for that year comparing between Scenarios 4 and 2 (Supplementary **Table 27.**). This means that including the cost of equipment upgrades, households will save money on average from installing more efficient appliances in the home. With current electricity prices, all efficiency upgrades are cost savings with the exception of water heaters. In our model, the CCE is 23.8 2010¢/kWh, which is greater than the current electricity price of 19.6 2010¢/kWh. This is driven by the high cost of solar water heating and heat pumps relative to standard electric water heaters, and the high penetration of these technologies in our model. If subsidies were employed, it could make these cost-neutral for the consumer, and greater market share of these alternate water heating technologies might also decrease the cost.

SUPPLEMENTARY DISCUSSION

There are multiple sources of uncertainty in our model which fall into either the category of 1) data uncertainty or 2) trend uncertainty. The sources of data uncertainty include elements within the base model or other projections that we use in our forecasts. Trend uncertainty includes the variability in future trends compared to what we've asserted in the model.

A primary source of data uncertainty is the GCM models. Variability exists from model to model within an RCP, and this variability in our forecast is captured in the figures presented in the main manuscript. Additionally, although our model is calibrated and validated, there is uncertainty in the archetype energy models due to 1) the internal uncertainty from the EnergyPlus model itself, and 2) the variability of buildings within an archetype category in LAC and the potential for an archetype to poorly represent some of those buildings. Another source of data uncertainty are the population projections for LAC.

Primary sources of trend uncertainty include consumer behavior, population growth, and appliance efficiency improvements. In this model, we assume that energy consumption in buildings is primarily linked to the building type, equipment, and weather conditions. We do not account for changes in behavior beyond our inclusion of 10% rebound, although there potentially could be significant shifts in energy consuming behavior, particularly around plug loads as small electronics use increases. Additionally, we assume that energy prices are stable relative to inflation, and therefore include no changes in consumption or fuel switching in response to market forces. The rates of technology adoption and saturation in our scenarios are based upon current market trends, but this could change significantly over the next 50 years. Population growth is being driven primarily by international immigration, and there is a large amount of uncertainty in that forecast. We assumed the population growth as constant for the purposes of our model, but there are many different potential paths for population increase in LAC which will have ramifications for building construction and energy use. Likewise, there are many potential future pathways for appliance efficiency improvements. Our study assumes a continuing trend of technological innovation combined with policy application, but both of these factors could be constrained in the future.

SUPPLEMENTARY REFERENCES

1. U.S. Department of Energy. *Water Heater Market Profile 2009*. (2009). https://www.energystar.gov/ia/partners/prod_development/new_specs/downloads/water_heaters/water_heater_market_profile_sept2009.pdf
2. U.S. Department of Energy. *Final Rule, Techincal Supporting Document Residential Refrigeration*. (2007).
3. National Academy of Sciences. *Real Prospects for Energy Efficiency in the United States*. (National Academies Press, 2010). doi:10.17226/12621 http://www.nap.edu/catalog/12621
4. Pacific Energy Center. *Pacific Energy Center's Guide to California Climate Zones and Bioclimatic Design*. (2006). http://www.pge.com/includes/docs/pdfs/about/edusafety/training/pec/toolbox/arch/climate/california_climate_zones_01-16.pdf
5. U.S. Bureau of Labor Statistics. Average Energy Prices, Los Angeles-Riverside-Orange County February 2016. (2016). Available at: http://www.bls.gov/regions/west/news-release/averageenergyprices_losangeles.htm. (Accessed: 12th April 2016) http://www.bls.gov/regions/west/news-release/averageenergyprices_losangeles.htm
